# ﻿A new species of *Cincticostella* Allen, 1971 (Ephemeroptera, Ephemerellidae) from Yunnan, China and establishment of a new species complex

**DOI:** 10.3897/zookeys.1205.125639

**Published:** 2024-07-01

**Authors:** Ye-Kang Sun, Rong-Long Yang, Zhi-Wei Tan, Xian-Fu Li, Luke M. Jacobus

**Affiliations:** 1 Institute of Eastern-Himalaya Biodiversity Research, Dali University, Dali 671000, Yunnan, China; 2 Yunnan Key Laboratory for Pollution Processes and Control of Plateau Lake-Watersheds, Yunnan Academy of Ecological and Environmental Sciences, Kunming 650034, Yunnan, China; 3 Collaborative Innovation Center for Biodiversity and Conservation in the Tree Parallel Rivers Region of China, Dali University, Dali, Yunnan, China; 4 Research Center of Ecology and Governance for Er’hai Lake Streams, Dali, Yunnan, China; 5 Division of Science, Indiana University Columbus, Columbus 47203, IN, USA

**Keywords:** Eastern-Himalaya, Ephemerellidae, Hengduan Mountains, Mayfly, Taxonomy

## Abstract

*Cincticostellajianchuan***sp. nov.** from Dali Bai Autonomous Prefecture, Yunnan Province, China, is described based on chorionic structure, nymph, and winged stages. The new species is closely related to *C.fusca* (Kang & Yang, 1995), but it can be distinguished in the male imago stage by its mesonotum and penes morphology, coloration, and the forking point of the stem of MA+Rs on the forewing; in the nymph stage, it can be distinguished by the length of the posterolateral projections of abdominal segment IX and the setation of the abdominal terga. Compared to other congeners, nymphs and male imagoes of the new species and *C.fusca* share several morphological characteristics, such as a larger body, mesothorax with medially notched anterolateral projections, forefemur without a subapical band of transverse spines of the nymphs, the area between C, Sc and R1 of the forewings distinctly pigmented, and an apical sclerite on the ventral face of the penes of the male imagoes, supporting the proposition of a new species complex, the *jianchuan* complex. The systematics of *Cincticostella* and related genera are discussed briefly.

## ﻿Introduction

The genus *Cincticostella* Allen, 1971 (Ephemeroptera: Ephemerellidae: Ephemerellinae: Ephemerellini) includes 22 species from throughout the eastern Palearctic and Indomalayan regions (Auychinda et al. 2022). The distinctive nymphs have the anterolateral angles of the prothorax projecting anteriorly and have a pair of large, wide, mesothoracic anterolateral processes (Jacobus and McCafferty 2008; Xie et al. 2009). Recent years have seen increased study of this genus, with nine of its species being described for the first time in the last five years (Martynov et al. 2019, 2021; Auychinda et al. 2020a, 2022). Also, several male adults have been described for the first time (Zhang et al. 2020, 2021; Zheng and Zhou 2021), resulting in a better understanding of this and related genera.

Due to uncertain relationships of species in the genus *Cincticostella* and possible polyphyly, the term “complex” has been used at various times to indicate distinctly different groups of species (Allen 1975; Martynov et al. 2019, 2021). According to Allen (1975), representatives of the *nigra*-group lack head tubercles and their middle and hind pairs of femora are narrow, not enlarged and the margins are without serration; ones of the *insolta*-group have suboccipital head tubercles and the middle and hind pairs of femora being enlarged with serrated margins and/or protuberances. But *C.gosei* (Allen, 1975) did not fit into either of these two complexes (Martynov et al. 2021). Therefore, the monotypic *C.gosei* complex was proposed by Martynov et al. (2021), according to *C.gosei* had a combination of characters. Up to now, the genus *Cincticostella* has three complexes. Martynov et al. (2021) and Auychinda et al. (2022) had reviewed the species included in each complex. The *nigra* complex is the largest, with 14 species (Martynov et al. 2021; Auychinda et al. 2022). Zhang et al. (2020) described the male imago of *C.fusca* Kang & Yang, 1995, a species within this group. They remarked about the unique form of the genitalia, but they did not recognize a new species complex for it. Li et al. (2020) noted a novel gene arrangement pattern in its mitochondrial genome.

During our recent survey of the mayfly fauna of the Hengduan Mountains area, at the eastern end of the Himalayas, a not yet described species of *Cincticostella* similar to *C.fusca* was found in Jianchuan County, Dali Bai Autonomous Prefecture, western Yunnan, China, at an altitude of more than 2200 m. Here, we describe this new *Cincticostella* species based on imago, subimago, nymph, and chorionic structure. Based on these new data and previous data for *C.fusca* (Zhang et al. 2020), we propose a new species complex within *Cincticostella*.

## ﻿Material and methods

*Cincticostella* nymphs were collected with a D-frame net from the riffle and running habitats of the Jinlong River, in Jianchuan County, Dali Bai Autonomous Prefecture, western Yunnan, China. Following the guidelines of Li et al. (2022) and Yang et al. (2023), the habitat photographs were taken using the mobile phone equipped with a Kase 40–75 mm macro lens. Water pH was measured with a YSI Professional Plus Multiparameter. Some specimens were dissected under a stereomicroscope and were mounted on slides with Hoyer’s Solution for examination under higher light magnification. Slide-mounted specimens were examined, photographed and measured under a Keyence VHX-S550E digital microscope. Eggs were dissected from female imagoes. Eggs were dried, coated with gold, observed and photographed by scanning electron microscopy (SEM). The final plates were prepared with Adobe Photoshop CC 2018.

All imagoes were collected by rearing the mature nymphs in the laboratory. All materials are stored in 95% ethanol. Holotype and paratype specimens are deposited in the Museum of Biology, Institute of Eastern-Himalaya Biodiversity Research, Dali University (MBDU). Species hypotheses utilize a morphological species concept.

## ﻿Results

**Ephemeroptera Latreille, 1810**

**Ephemerellidae Klapálek, 1909**

### ﻿ Cincticostella jianchuan sp. nov.

Taxon classificationAnimaliaEphemeropteraEphemerellidae

345BB749-8770-5F59-8F68-17C82B531ADC

https://zoobank.org/87E98558-629C-4512-8224-A2BD9C29791C

Figs 1 , 2 , 3 , 4 , 5 , 6 , 7 , 8 , 9 , 10 , 11 , 12 , 13 , 14 , 15 , 16 , 17 , 18 , 19 , 20

#### Material examined.

***Holotype***: male imago, with final nymphal instar exuviae (in ethanol), China, Yunnan Province, Dali City, Jianchuan County, Jinlong River, 26°35'2.7"N, 99°51'45.0"E, 2371 m a.s.l., 09.V.2022, coll. Xian-Fu Li and Rong-Long Yang. ***Paratypes***: 40 nymphs, 24 imagos and 20 subimagos reared from nymphs with same data as holotype.

#### Diagnosis.

The new species is similar to *C.fusca*. These two species can be differentiated from other *Cincticostella* species by the following combination of characters in the nymph: 1) caudal filaments length subequal to or slightly shorter than body length, 2) genae developed into obvious extensions, 3) mesothoracic projection with notch, 4) forefemur without transverse row of setae on upper surface, and 5) all articulations of caudal filaments with spines; and by the following combination of characters in the male imago: 1) area between C, Sc and R1 of forewings colored, 2) styliger plate with median convex lobe-like posterior projection, and 3) general shape of penes, especially the form of the apical sclerite on the ventral face.

The new species can be distinguished from *C.fusca* in the male imago stage by its coloration, its mesonotal scutellum morphology, and by the shape of genitalia. In the nymphal stage, the two species can be distinguished by the setation of abdominal terga and the shapes of posterolateral projections of tergum IX. Specifically:

1. Cells of costal and subcostal fields of the forewing of *C.jianchuan* sp. nov. are brown (Figs 7E, 10D, 17A, B), whereas these cells are dark brown in *C.fusca* (Zhang et al. 2020: fig. 4A, B).
2. The mesonotum of *C.jianchuan* sp. nov. clearly has three projections on the posterior margin (Fig. 8A), while *C.fusca* has only two projections apparent (Zhang et al. 2020: fig. 4D).
3. *Cincticostellajianchuan* sp. nov. has two subapical hemispherical grooves on the ventral face of the penes (Fig. 9B, E), but *C.fusca* has two additional large ventral projections on the upper middle of the ventral face of the penes (Zhang et al. 2020: fig. 5C, E).
4. In terms of wing venation, the stem of the MA+Rs fork of *C.jianchuan* sp. nov. occurs at a slightly more distal location than the fork of MP (Fig. 7E). However, in *C.fusca*, the MP fork and the stem of the MA+Rs fork are equidistant from the base of the wings to the margin (Zhang et al. 2020: fig. 6A).
5. The posterolateral projections of abdominal segment IX of the nymph of *C.jianchuan* sp. nov. (Fig. 5A, C, D) are longer than the same posterolateral projections on *C.fusca* (Kang and Yang 1995: fig. 3H).
6. Also on the nymph, each lateral margin of abdominal segments IV–VIII and median area of terga II–X of *C.fusca* nymph has setae (Kang and Yang 1995: fig. 3G). In contrast, the nymph of *C.jianchuan* sp. nov. lacks setae in the corresponding locations (Fig. 5A, B).

#### Descriptions.

**Figure 1.**
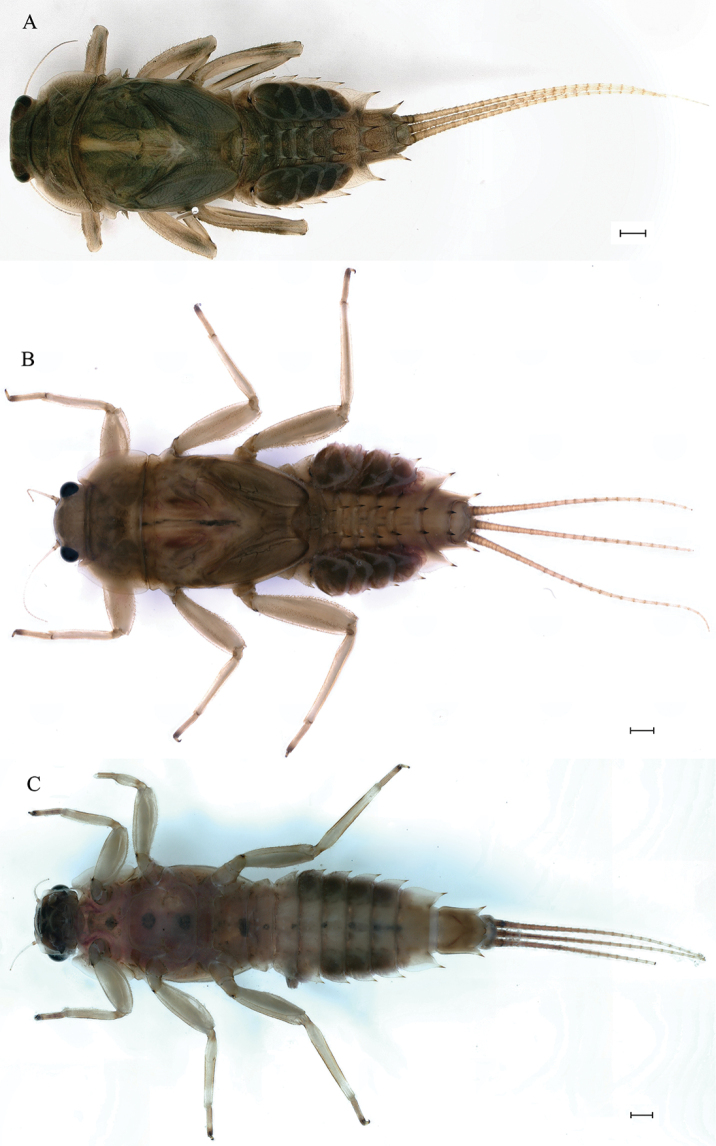
Last nymphal instar of *Cincticostellajianchuan* sp. nov. **A** dorsal habitus of male **B** dorsal habitus of female **C** ventral habitus of male. Scale bar: 1000 µm (**A, B**).

***Last instar nymph*** (in alcohol). Last instar nymph: head width, male 3.1–3.3 mm; female 3.4–3.5 mm; body length (excluding tails), male 15.3–16.7 mm, female 18.0–18.9 mm; cerci length, male 8.9–11.6 mm, female 10.5–12.8 mm, middle caudal filament, male 9.3–10.9 mm, female 10.6–11.5 mm. Body color reed green to brown (Fig. 1A–C).

**Figure 2.**
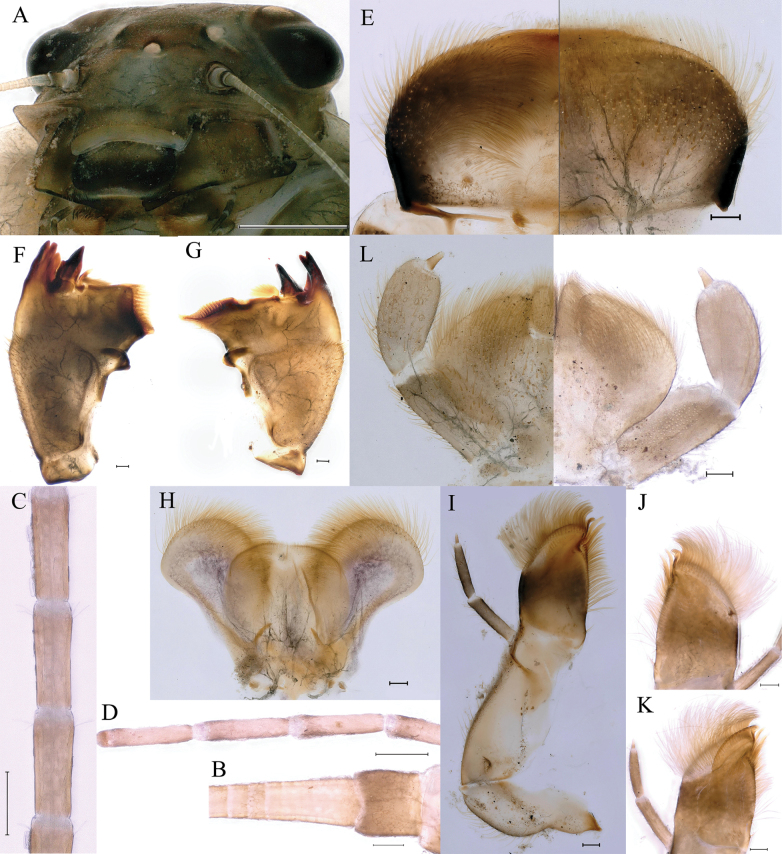
Nymphs of *Cincticostellajianchuan* sp. nov. **A** head **B** basal part of antenna **C** middle part of antenna **E** apical part of antenna **E** labrum **F** left mandible **G** right mandible **H** hypopharynx (ventral view) **I** left maxilla **J** apex of right maxilla (dorsal view) **K** apex of right maxilla (ventral view) **L** labium (ventral view). Scale bar: 1000 μm (**A**); 100 μm (**B–K**).

***Head***: base color reed green to brown, genae expanded into lobes (Fig. 2A); middle portion of antennae with tiny setae on articulations (Fig. 2C), basal (Fig. 2B) and apical (Fig. 2D) portions of antennae without such setae. Labrum width equal to about 1/3 head width, setae on dorsal and ventral surfaces; those on ventral surface and anterolateral margin relatively longer and more densely arranged (Fig. 2E). Mandible: both mandibles with numerous short hair-like setae on dorsal and lateral surfaces; trifurcated outer incisor and bifurcated inner incisor (Fig. 2F, G). Left mandible without seta near mola, prostheca comprised of two tufts of spines with common stem (Fig. 2F). Right mandible with row of irregular hair-like setae under mola, prostheca with one tuft of hair-like setae on common base (Fig. 2G). Hypopharynx (Fig. 2H): lingua recumbent oval, with shallow anteromedian concavity, short setae densely situated on anterolateral margins, apical 1/4 with pale spot; superlinguae with slight curved outer, anterior margin and surface densely covered with long setae. Maxilla (Fig. 2I–K): apex with 2 large canine teeth and slightly curved plate, apical margin of plate crenate (Fig. 2K); tuft of stout setae at apex; galea-lacinia with several setae near base; lateral margins of stipes and cardo with fine setae; three-segmented maxillary palp covered with hair-like setae and sharp setae, segment III very small and short, segment length ratio from base to apex = 5.3: 4.7: 1.0 (Fig. 2I–K). Labium (Fig. 2L): paraglossae semicircular, apical half of dorsum with closely set sharp setae; apical three quarters of venter with loose set of fine setae; glossae long and ellipsoid with setae; labial palp with 3 segments, surfaces of segments I and II with sharp setae; segment II slightly enlarged; segment III very small, segment length ratio from base to apex = 7.2: 5.1: 1.0.

**Figure 3.**
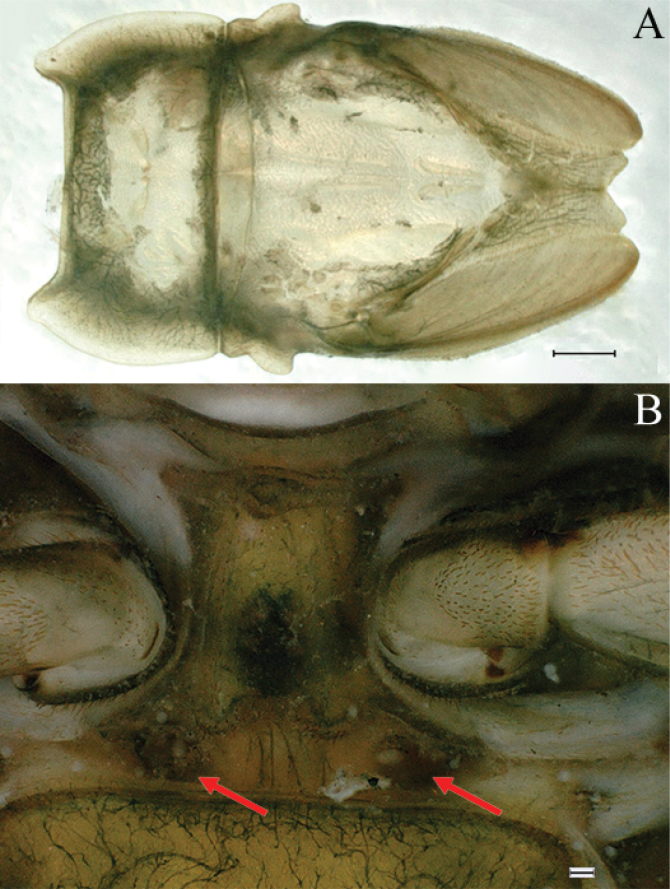
Nymphs of *Cincticostellajianchuan* sp. nov. **A** thorax of last nymphal instar (dorsal view) **B** ventral view of pronotum with hole-like structures. Scale bar: 1000 μm (**A**); 500 μm (**B**).

**Figure 4.**
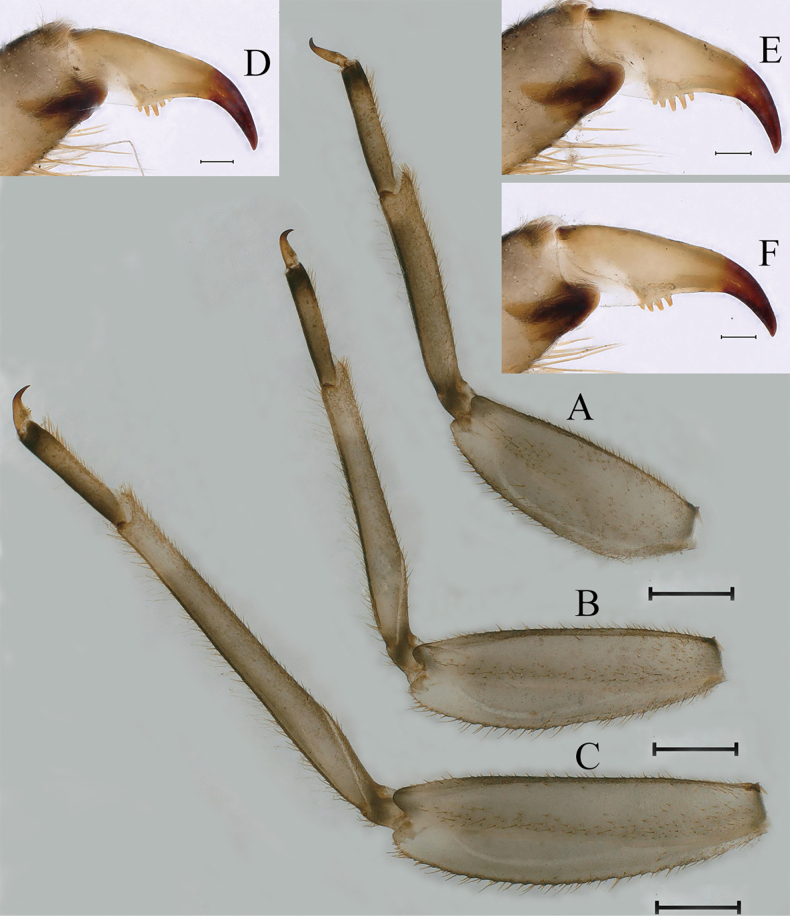
Nymphs of *Cincticostellajianchuan* sp. nov. **A** foreleg **B** midleg **C** hindleg **D** claw of foreleg **E** claw of midleg **F** claw of hindleg. Scale bar: 1000 μm (**A–C**); 100 μm (**D–F**).

***Thorax*** general color yellowish to reed green. Pronotum with paired small brownish green irregular stripes, anterolateral corners produced anteriorly (Fig. 1A, B); mesonotum with medial pale stripe, paired small brownish green dots and irregular stripes (Fig. 1A, B), lateral margins each with blunt projection near anterolateral corner (Figs 1A, B, 3A). Prosternum with pair of hole-like structures (Fig. 3B, indicated by black arrow). Legs: posterolateral angles of fore and middle coxae each with acute projection (Fig. 1A). Dorsal surface of femur of each leg with setae on median, posterior and anterior areas (Fig. 4A–C), ratio of overall femur width of foreleg: middle-leg: hindleg = 1.0: 1.0: 1.1. Ratio of femur: tibia: tarsus of foreleg = 1.9: 1.8: 1.0, ratio of femur: tibia: tarsus of middle-leg = 2.4: 2.4: 1.0, ratio of femur: tibia: tarsus of hindleg = 2.6: 3.0: 1.0. Claws pale, apices dark brown, with 4–5 blunt denticles medially (Fig. 4D, E).

**Figure 5.**
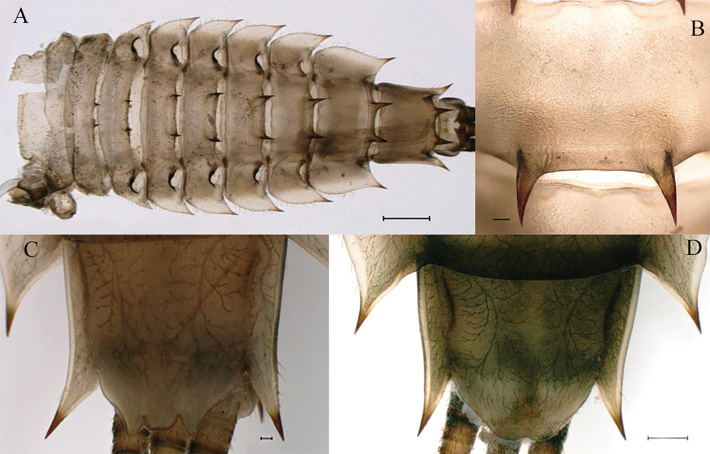
Nymphs of *Cincticostellajianchuan* sp. nov. **A** abdomen (dorsal view) **B** abdominal terga VII enlarged **C** posterior part of abdomen of male (ventral view) **D** posterior part of abdomen of female (ventral view). Scale bar: 1000 μm (**A**); 500 μm (**D**); 100 μm (**B, C**).

**Figure 6.**
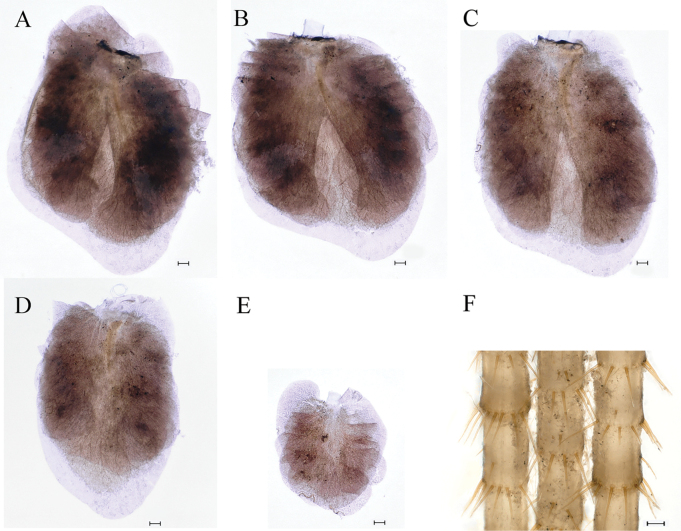
Nymphs of *Cincticostellajianchuan* sp. nov. **A** gill III **B** gill IV **C** gill V **D** gill VI **E** gill VII **F** caudal filaments. Scale bar: 100 μm (**A–F**).

***Abdomen***: abdominal segments II–IX each with posterolateral projection with clavate setae; largest posterolateral projection on segment VIII, long and divergent posterolateral projections on segment IX (Fig. 5A). Posterior margins of segments II–IX each with pair of sharp tubercles, progressively longer on segments II–VIII (Fig. 5A). Each tergum without setae on posterior margin and dorsal area (Fig. 5A, B), but posterior margin of tergum I with long fine setae. Posterior margin of sternum IX of male wavy (Fig. 5C); posterior margin of sternum IX of female concave (Fig. 5D). Gills III–V subequal in size, dorsal lamellae oblique oval, ventral lamellae each with deep cleft (Fig. 6A–C); gill VI dorsal lamella rounded, ventral lamella without deep cleft (Fig. 6D); gill VII small, somewhat heart-shaped but rounded (Fig. 6E). Distal part of caudal filaments darkly colored, each segment with whorled acute setae at apex and few sharp setae near middle area, median segments with long fine setae on lateral margins loosely arranged (Fig. 6F).

**Figure 7.**
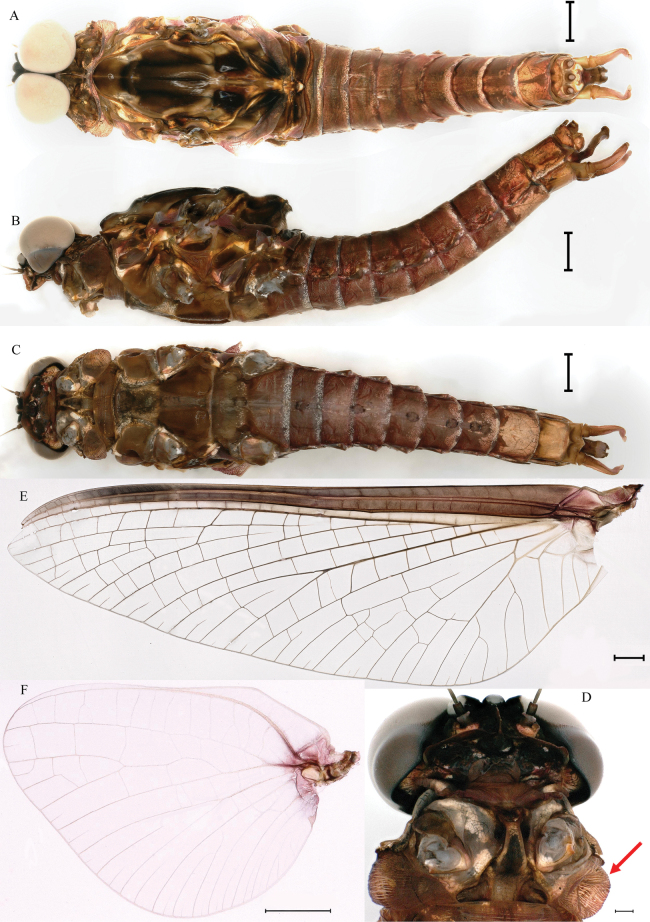
Male imago of *Cincticostellajianchuan* sp. nov. **A** dorsal view of body **B** lateral view of body **C** ventral view of body **D** ventral view of head and pronotum, with air sac-like structure indicated by red arrow **E** forewing **F** hindwing. Scale bar: 1000 μm (**A–F**).

***Male imago*** (in ethanol). Body length 18.3–18.8 mm (excluding tails), head width 3.1–3.3 mm, forewing length 19.4–20.0 mm, hindwing length 5.4–5.9 mm, cerci length 8.9–11.6 mm, middle caudal filament 9.3–10.9 mm. Body color brown to dark brown (Figs 7A–C, 17A).

***Head*.** Compound eyes contiguous, upper portion brown and lower portion black (Fig. 7B, D).

**Figure 8.**
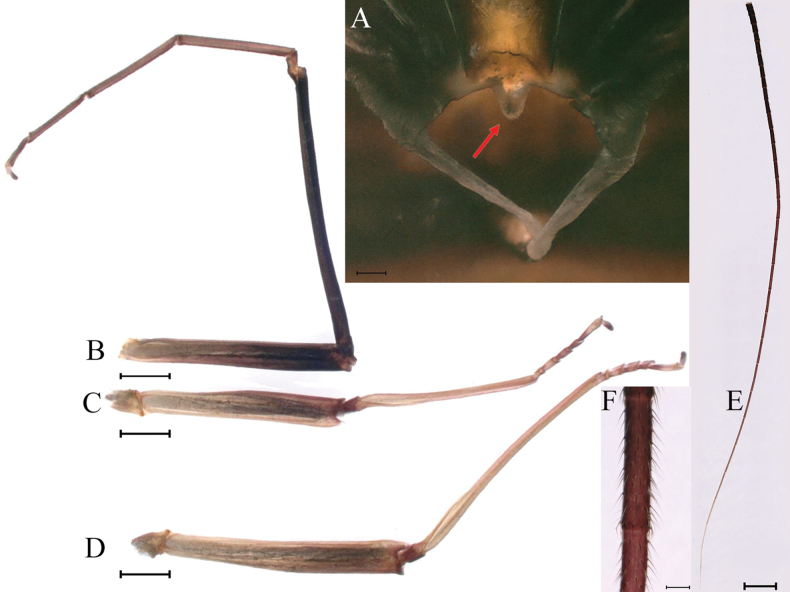
Male imago of *Cincticostellajianchuan* sp. nov. **A** lateral scutellar projections, middle one indicated by red arrow **B** foreleg **C** midleg **D** hindleg **E** cerci **F** cerci enlarged. Scale bar: 100 μm (**A, F**); 1000 μm (**B–E**).

***Thorax*.** Pronotum with expanded posterolateral air sac-like structure (Fig. 7A–D, indicated by red arrow). Mesonotum with three projections on posterior margin, middle projection short (Fig. 8A, indicated by red arrow). Forewings generally hyaline, veins brown; all cells of costal and subcostal fields tinted with brown, 2/3 cells from base to margin of subcostal and radial fields tinted with brown; cross veins in stigmatic area slightly oblique, and those between costal and subcostal areas separated into two rows of cells. MA forked 2/3 of distance from base to margin; stem of MA+Rs fork at very base, just slightly more distal than fork of MP (Fig. 7E). Hindwing totally hyaline, leading margin slightly concave; MA single, MP forked symmetrically (Fig. 7F). Forelegs brown to dark brown (Fig. 8B), mid- and hindlegs brown (Fig. 8C, D). Femur: tibia: tarsus of foreleg = 1.0: 1.3: 1.6, tarsal segments from basal to apical = 1.0: 5.4: 5.0: 3.5: 1.8; femur: tibia: tarsus of midleg = 2.2: 2.3: 1.0, tarsal segments arranged in decreasing order = 1.0: 1.4: 1.3: 1.0: 2.5; femur: tibia: tarsus of hindleg = 2.6: 3.0: 1.0, tarsal segments arranged in decreasing order = 1.0: 1.3: 1.5: 1.1: 2.9. Claws of all legs similar, one blunt and one hooked.

***Abdomen*.** Terga II–V each with pale stripe on posterior margin, pair of diffuse dark dots near posterior margins of terga II–IX; diffuse dark stripes on middle and lateral faces of terga VIII–IX (Fig. 7A, B). Dark ganglionic marks on sterna I–VII (Fig. 7C). Small portion of sternum VII and most of sterna VIII–IX light colored (Fig. 7C). Styliger plate with middle projection (Fig. 7C). Caudal filaments brown to dark brown, with spines (Fig. 8E, F).

**Figure 9.**
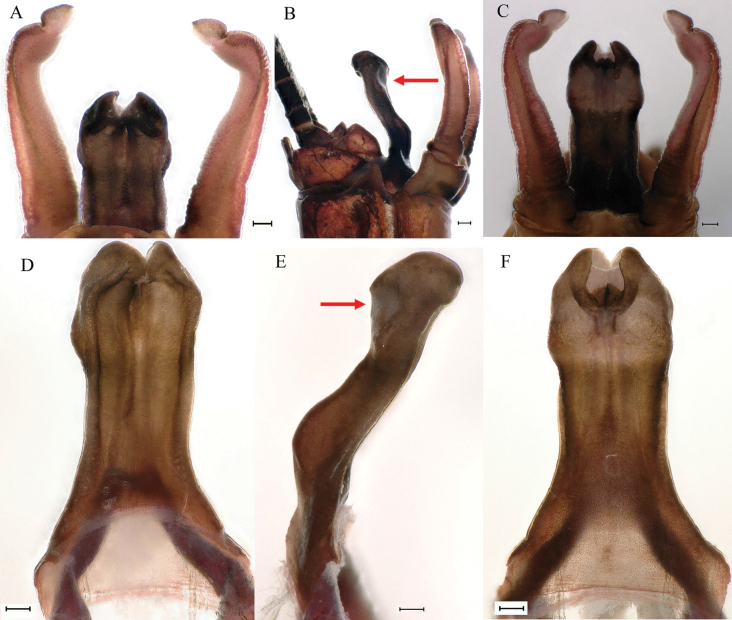
Male imago of *Cincticostellajianchuan* sp. nov. **A** genitalia (dorsal view) **B** genitalia (lateral view) with groove indicated by arrow **C** genitalia (ventral view) **D** penes (dorsal view) **E** penes (lateral view), with position of groove indicated by arrow **F** penes (ventral view). Scale bar: 100 μm (**A–F**).

***Genitalia*.** Forceps covered with stout setae; segment 3 globular; segment 2 angled inward distally and with slight subapical constriction (Fig. 9A–C). Penis lobes compact, with linear groove on apical 1/2 of dorsal face (Fig. 9A, D), apical sclerite (Fig. 9C, F), two subapical hemispherical grooves (Fig. 9B, E, indicated by red arrows), one basal obvious bump (Fig. 9B, E), one subapical large pale plate on ventral face (Fig. 9C, F), lobes separated by slight cleft (Fig. 9C, F).

**Figure 10.**
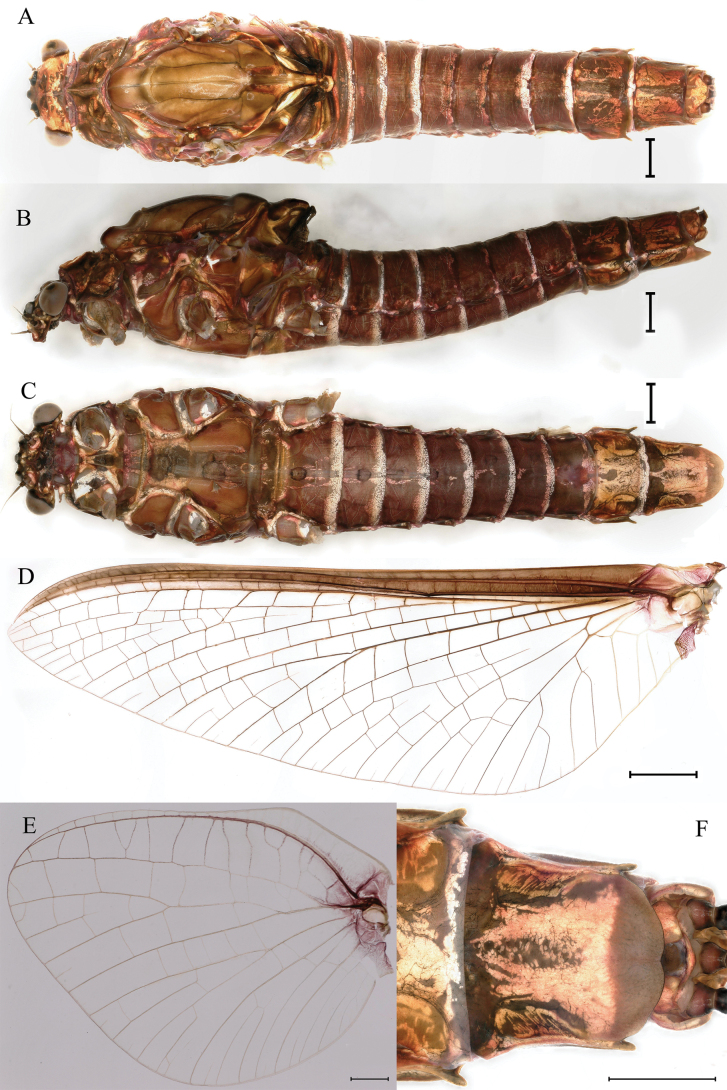
Female imago of *Cincticostellajianchuan* sp. nov. **A** dorsal view **B** lateral view **C** ventral view **D** forewing **E** hindwing **F** posterior part of abdomen (ventral view). Scale bar: 2000 μm (**D**); 1000 μm (**A–C, F**); 500 μm (**E**).

**Figure 11.**
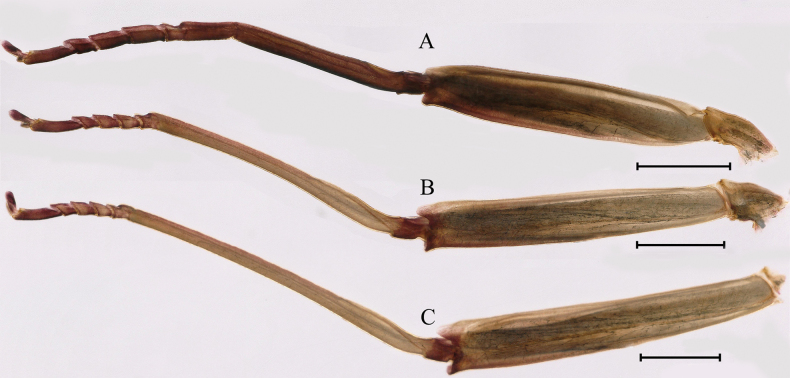
Female imago of *Cincticostellajianchuan* sp. nov. **A** foreleg **B** midleg **C** hindleg. Scale bar: 1000 μm (**A–C**).

***Female imago*** (in ethanol). Color pattern similar to male (Figs 10A–F, 11 A–C, 17B); body length 19.1–22.9 mm (excluding tails), head width 2.9–3.5 mm, cerci length 17.6–23.7 mm, middle caudal filament 17.3–22.1 mm, forewing 19.9–23.4 mm, hindwing 5.5–6.1 mm. Lengths of femur: tibia: tarsus of foreleg = 1.9: 1.6: 1.0, tarsal segments from basal to apical = 1.0: 2.0: 1.7: 1.1: 2.3; femur: tibia: tarsus of midleg = 2.4: 2.4: 1.0, tarsal segments from basal to apical = 1.0: 1.2: 1.2: 1.1: 3.2; femur: tibia: tarsus of hindleg = 2.7: 3.1: 1.0, tarsal segments from basal to apical = 1.0: 1.1: 1.4: 1.0: 2.7. Compared with male, pronotum with nonexpanded posterolateral air sac-like structures; inner margin of femur of foreleg covered with spines. Posterior margin of subgenital plate produced to 1/5 length of sternum VIII. Posterior margin of subanal plate with obvious median cleft (Fig. 10F). Color pattern of caudal filaments similar to male.

**Figure 12.**
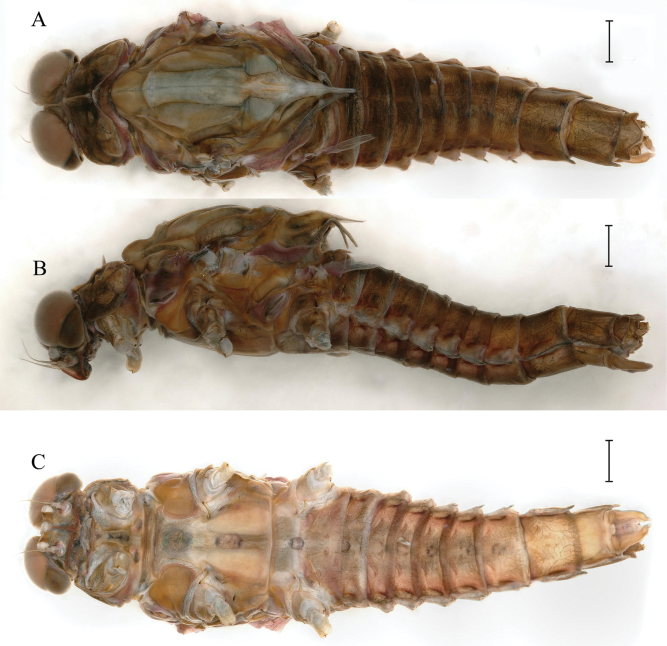
Male subimago of *Cincticostellajianchuan* sp. nov. **A** dorsal view **B** lateral view **C** ventral view. Scale bar: 1000 μm (**A–C**).

**Figure 13.**
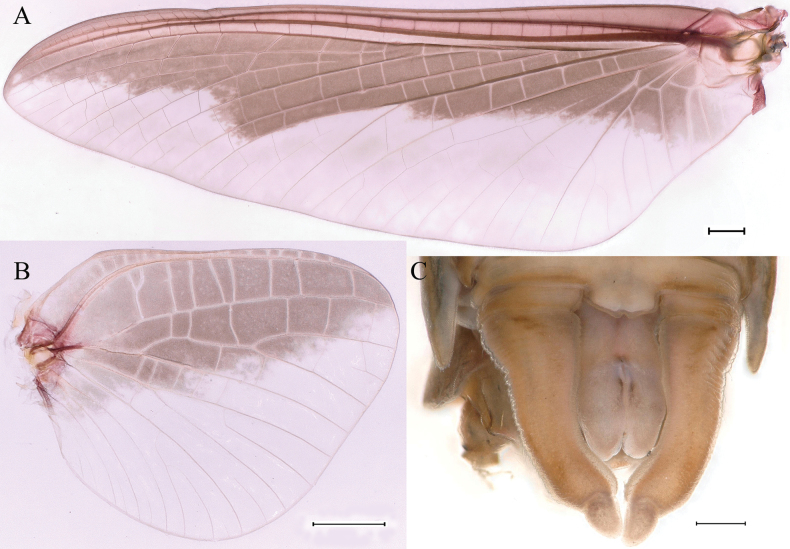
Male subimago of *Cincticostellajianchuan* sp. nov. **A** forewing **B** hindwing **C** ventral view of genitalia. Scale bar: 1000 μm (**A, B**); 200 μm (**C**).

**Figure 14.**
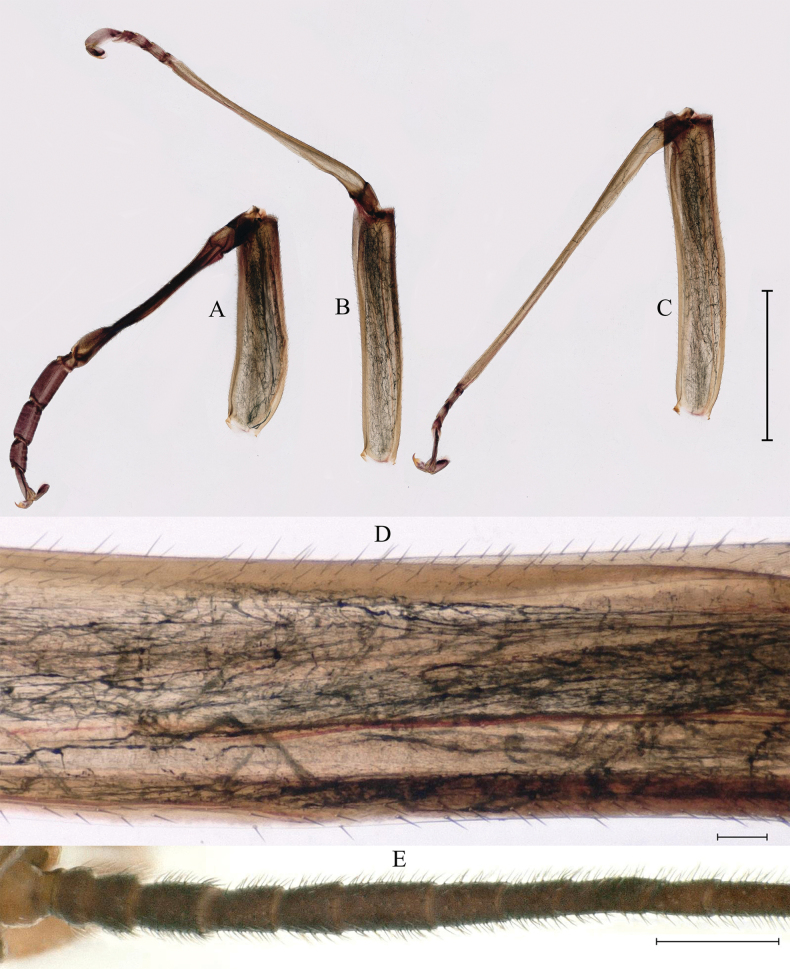
Male subimago of *Cincticostellajianchuan* sp. nov. **A** foreleg **B** midleg **C** hindleg **D** closer view of femur of midleg **E** cerci. Scale bar: 2000 μm (**A–C**); 500 μm (**E**); 100 μm (**D**).

***Male subimago*.** Body color taupe gray to dark brown (Figs 12A–C, 17B). Front portions of forewing and hindwing taupe gray and rear portions nearly white (Fig. 13A, B). Pronotum with nonexpanded posterolateral air sac-like structures; scutellum with three long, pointed posterior prolongations (Fig. 12A, B). Caudal filaments with relative densely spines (Fig. 14E). Apical sclerite of penes incomplete (Fig. 13C). Body length 13.0–14.5 mm (excluding tails), head width 2.6–3.0 mm, cerci length 8.0–16.5 mm, middle caudal filament 8.5–16.4 mm, forewing 19.4–19.8 mm, hindwing 4.6–5.1 mm (Fig. 12A–C, 13A–C, 14A–E). Margins of femur and tibia of foreleg, midleg and hindleg all densely covered with spines (Fig. 14A–D). Length of femur: tibia: tarsus of foreleg = 1.3: 1.4: 1.0, tarsal segments from basal to apical = 1.0: 2.7: 2.5: 1.8: 2.2; femur: tibia: tarsus of midleg = 2.4: 2.3: 1.0, tarsal segments from basal to apical = 1.2: 1.7: 1.3: 1.0: 2.9; femur: tibia: tarsus of hindleg = 3.1: 3.6: 1.0, tarsal segments from basal to apical = 1.0: 3.0: 3.0: 2.0: 6.2.

**Figure 15.**
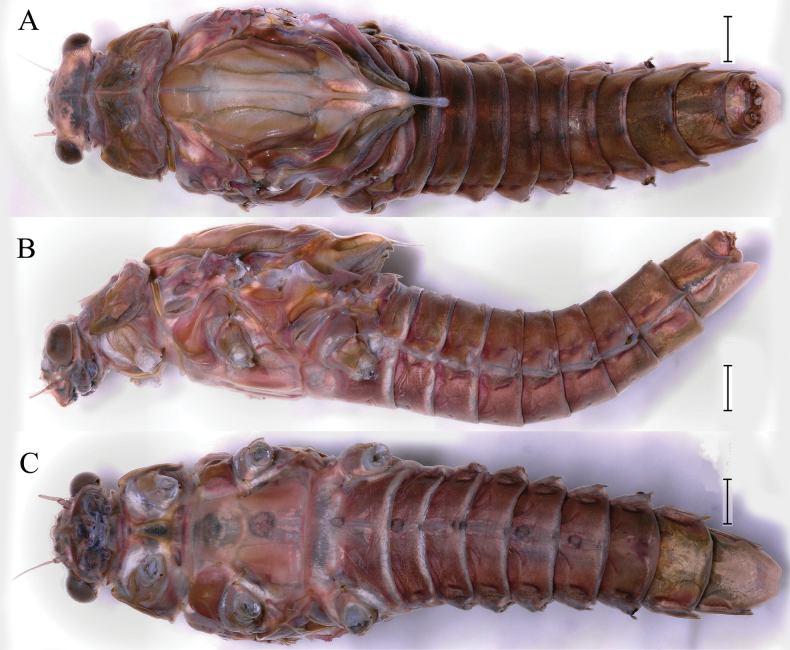
Female subimago of *Cincticostellajianchuan* sp. nov. **A** dorsal view **B** lateral view **C** ventral view. Scale bar: 1000 μm (**A–C**).

**Figure 16.**
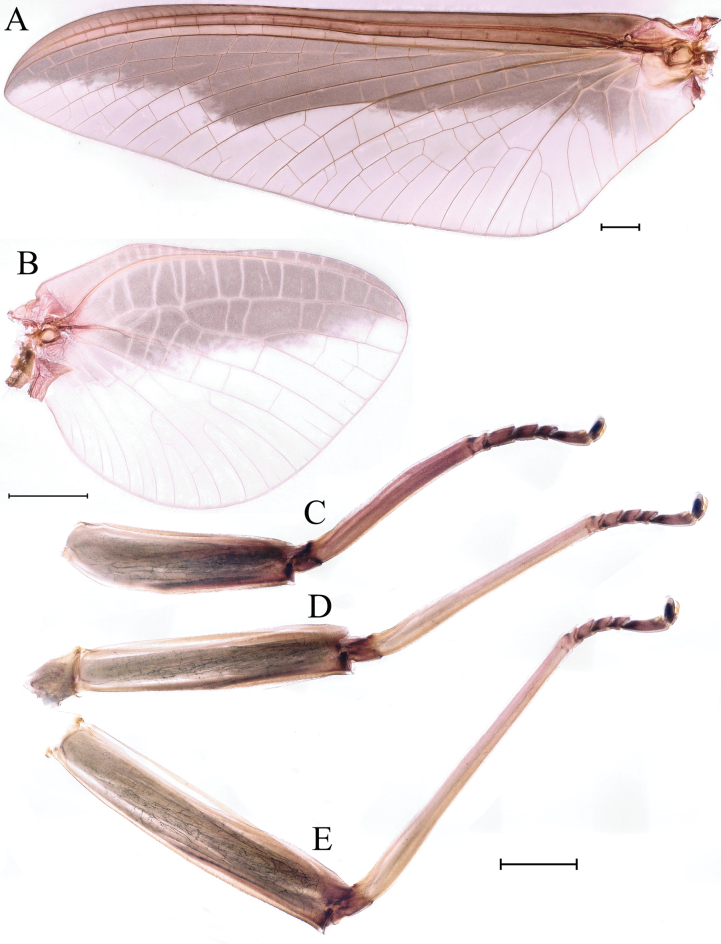
Female subimago of *Cincticostellajianchuan* sp. nov. **A** forewing **B** hindwing **C** foreleg **D** midleg **E** hindleg. Scale bar: 1000 μm (**A–E**).

**Figure 17.**
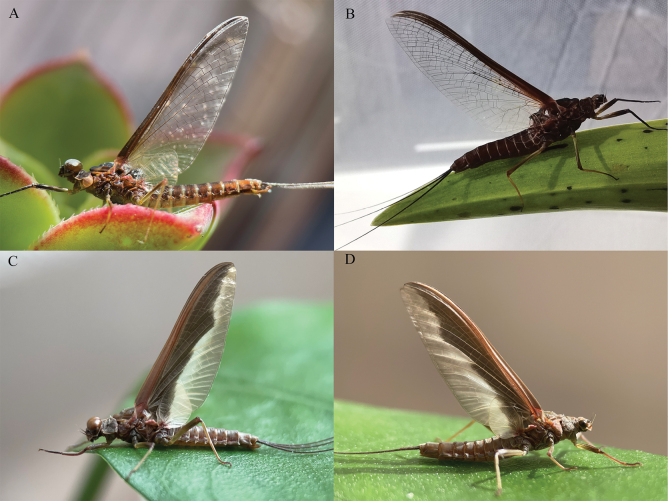
Winged stages of *Cincticostellajianchuan* sp. nov. (living) **A** male imago **B** female imago **C** male subimago **D** female subimago.

***Female subimago*** (in alcohol). Similar to male subimago except for usual sexual differences (Figs 15A–C, 16A–E, 17C). Length of femur: tibia: tarsus of foreleg = 1.7: 1.6: 1.0, tarsal segments from basal to apical = 1.0: 1.7: 1.4: 1.0: 2.1; femur: tibia: tarsus of midleg = 2.4: 2.5: 1.0, tarsal segments from basal to apical = 1.0: 1.3: 1.3: 1.2: 2.8; femur: tibia: tarsus of hindleg = 3.1: 3.6: 1.0, tarsal segments from basal to apical = 1.0: 1.7: 1.4: 1.3: 3.2. Inner margins of tarsus of foreleg, midleg and hindleg densely covered with spines (Fig. 8D–F, J). Head width 2.9–3.6 mm, body length 17.5–19.7 mm (excluding tails), forewing length 18.6–22.2 mm, hindwing length 3.8–5.7 mm, cerci length 12.2–13.8 mm, middle caudal filament 13.0–13.8 mm.

**Figure 18.**
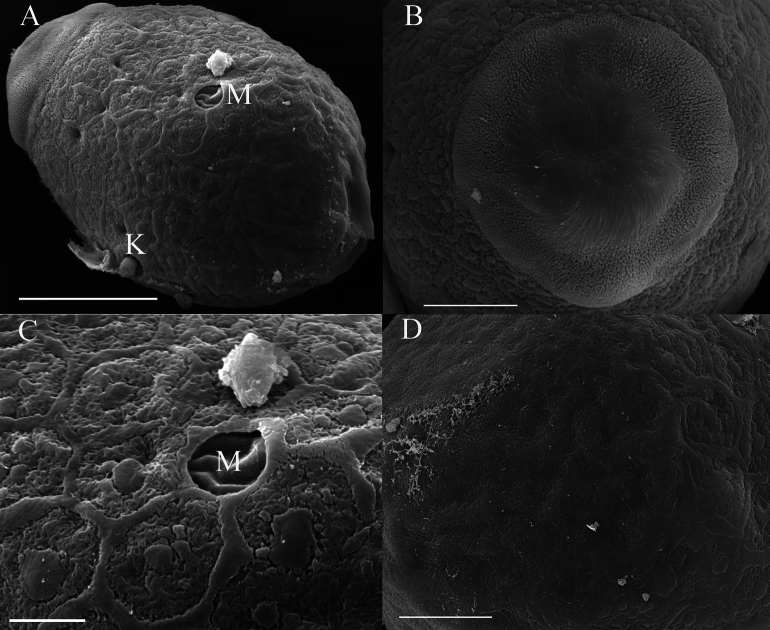
Egg of *Cincticostellajianchuan* sp. nov. **A** lateral view with micropyle (M) and knob of attachment structure (K) **B** polar cap **C** micropyle (M) enlarged **D** bottom view. Scale bar: 50 μm (**A**); 20 μm (**B–D**); 10 μm (**C**).

***Eggs***: dissected from female imago. Length 142–207 μm, width 96–120 μm. Ovoid with polar cap composed of dense filaments, each filament with intumescent terminal (Fig. 18A, B). Chorion with irregular polygonal ridges except subpolar areas (Fig. 18A–D); mesh with variety of tubercles medially; knobs of attachment structure and micropyle distributed near equator (Fig. 18A, C).

#### Persistent mouthparts of winged stages.

**Figure 19.**
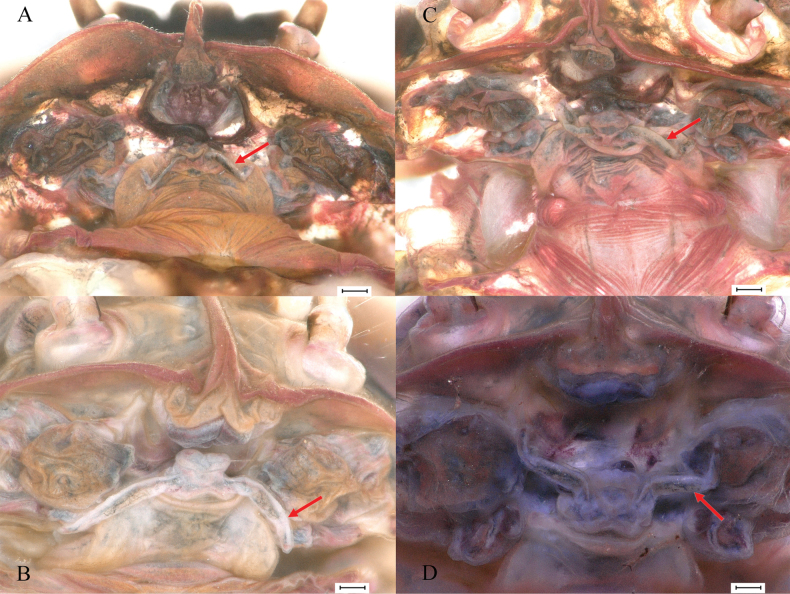
Persistent mouthparts of *Cincticostellajianchuan* sp. nov. Labium indicated by arrow. **A** male imago **B** female imago **C** male subimago **D** female subimago. Scale bars: 100 μm (**A–D**).

The new species presents persistent but vestigial mouthparts in the winged stages; in ventral view of head, the labium is present and clearly visible (Fig. 19A–D, indicated by red arrow).

#### Etymology.

The specific epithet is named after the type locality, Jianchuan County, Dali Bai Autonomous Prefecture, Yunnan Province, China. The English common name of this species is the Jianchuan spiny crawler mayfly. The Chinese name is 剑川带肋蜉.

#### Distribution.

Yunnan, China.

#### Ecology.

**Figure 20.**
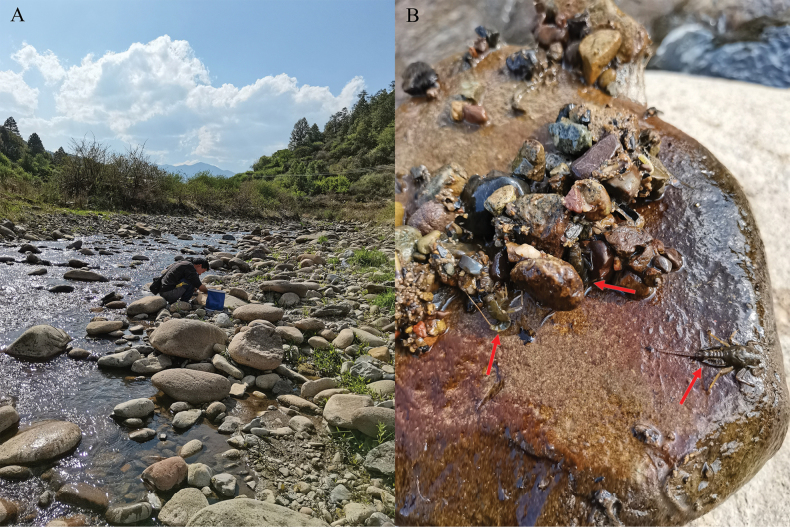
Habitat of *Cincticostellajianchuan* sp. nov. **A** general habitat of Jinlong River **B** nymphs on the cobblestone, indicated by arrows.

The nymphs of this new species were collected from upstream reaches of the Jinlong River in Jinchuan County, Dali Bai Autonomous Prefecture, Yunnan Province. The nymphs prefer run and riffle habitats containing stones of various sizes (Fig. 20A, B). Sampling sites were located at high altitudes (above 2300 m); the river and associated riparian zone passed through relatively natural habitat or traditional agriculture; width of the river was 3.0–9.7 m, but can reach 15 m during the flood season (Fig. 20A); the water quality tended to be alkaline (pH = 9.04). In the laboratory, the mature nymphs quickly completed the molting process on the water surface from 9 pm to 12 pm at night. The subimago stage persisted until the third noon or afternoon, with the observed lifespan of imagoes being about 3–4 days.

## ﻿Discussion

As discussed to some extent in the diagnosis section, *C.jianchuan* sp. nov. is closely related to *C.fusca*, whose nymph and imago share the following characters. In the nymph: 1) larger body size; 2) a pair of hole-like structures on the prosternum (Fig. 3B); 3) mesothorax with medially notched anterolateral projections (Fig. 3A; Kang and Yang 1995: fig. 3B; Zhang et al. 2020: fig. 1C); 4) forefemur without a transverse band of setae on the upper surface (Fig. 4A; Kang and Yang 1995: fig. 3D; Zhang et al. 2020: fig. 3A); and 5) without serration of margins of middle femora (Fig. 4B; Zhang et al. 2020: fig. 3B) and hind femora (Fig. 4C; Zhang et al. 2020: fig. 3C). In the male imago: 1) larger body size; 2) area between C, Sc and R1 distinctly pigmented (Fig. 7E, 10D, 17A, B; Zhang et al. 2020: fig. 4A, B, 5A, 6A); and 3) with apical sclerite on ventral face of the penes (Fig. 9C, F; Zhang et al. 2020: fig. 5C, E, 6C, D).

*Cincticostellajianchuan* sp. nov. and *C.fusca* differ from all other described *Cincticostella* species in these combinations of characters, which offer support for the recognition of a new species complex, the *jianchuan* complex. Although representatives of the *jianchuan* complex fit into the *nigra*-group, according to Allen (1975), they lack head tubercles and their middle and hind pairs of femora are narrow, not enlarged, and the margins are without serrations. But through our field investigations of the central, southern, and southwestern regions of China, the following characteristics demonstrate their stable uniqueness. The body size of the *jianchuan* complex is larger than the other complexes of *Cincticostella*. Mesothoracic anterolateral projections of the *C.insolta* complex (such as Martynov et al. 2019: figs 3, 4), *C.nigra* complex (such as Martynov et al. 2021: fig. 1C, D) and *C.gosei* complex (Martynov et al. 2021: fig. 13C, D) are not notched. The forefemora of the *C.nigra* complex (such as Martynov et al. 2021: fig. 3A) and *C.gosei* complex (Zhang et al. 2021: fig. 2D) have subapical bands of transverse setae, and the *C.insolta* complex (such as Martynov et al. 2019: figs 15, 16) and *C.gosei* complex (Zhang et al. 2021: fig. 2E, F) have serrations on the margins of middle and hind femora. The area between C, Sc and R1 of the *C.insolta* complex (Zheng and Zhou 2021: fig. 4A), *C.nigra* complex (pers. obs., Xian-Fu Li) and *C.gosei* complex (Zhang et al. 2021: fig. 3A, B) are semihyaline. The *Cincticostellainsolta* complex (Zheng and Zhou 2021: fig. 6C), *C.nigra* complex (pers. obs., Xian-Fu Li) and *C.gosei* complex (Zhang et al. 2021: fig. 4F) male imagoes lack the apical sclerite on the ventral face of the penes. In addition, the forewing (Figs 13A, 16A, 17C) and the hindwing (Figs 13B, 16B, 17D) of the subimago of *C.jianchuan* sp. nov. have clear differences from the *C.insolta* complex (Zheng and Zhou 2021: fig. 4B), *C.nigra* complex (pers. obs., Xian-Fu Li) and *C.gosei* complex (Zhang et al. 2021: fig. 3C, D).

The *jianchuan* complex species are at least superficially similar to species currently placed in four other genera related to *Cincticostella*, which include *Adoranexa* Jacobus & McCafferty, 2008, *Ephacerella* Paclt, 1994, *Spinorea* Jacobus & McCafferty, 2008, and *Notacanthella* Jacobus & McCafferty, 2008, based on their large size, abdominal armature of nymphs, male imago genital forceps morphology and other features. All of the species in these five genera, except for the type species of *Notacanthella*, have nymphs with a denticulate blade on the apex of the maxilla (Jacobus and McCafferty 2008). Distinctions between these genus groups (Jacobus and McCafferty 2008) were based mostly on exact combinations of nymphal thoracic projections and questionable qualities of the maxillary blade, at least some of which have been shown to be unreliable (Auychinda et al. 2020b). Zhang et al. (2021) also showed that some species based on male adults had been incorrectly assigned to genus. These facts, along with the number of species in the genus *Cincticostella* nearly doubling in very recent years, would suggest that systematics of this genus complex and the specific composition of various nominal genus groups (including their current junior synonyms) should be re-evaluated based on morphology and perhaps also with molecular data (Ogden et al. 2009).

## Supplementary Material

XML Treatment for Cincticostella jianchuan

## ﻿Additional information

### Author ORCIDs

Xian-Fu Li https://orcid.org/0000-0002-1258-1573

Luke M. Jacobus https://orcid.org/0000-0002-7201-3922

### Data availability

All of the data that support the findings of this study are available in the main text.
